# Characterization of *Scedosporium apiospermum* Glucosylceramides and Their Involvement in Fungal Development and Macrophage Functions

**DOI:** 10.1371/journal.pone.0098149

**Published:** 2014-05-30

**Authors:** Rodrigo Rollin-Pinheiro, Livia Cristina Liporagi-Lopes, Jardel Vieira de Meirelles, Lauro M. de Souza, Eliana Barreto-Bergter

**Affiliations:** 1 Departamento de Microbiologia Geral, Instituto de Microbiologia Professor Paulo de Góes, Universidade Federal do Rio de Janeiro, Rio de Janeiro, Rio de Janeiro, Brazil; 2 Departamento de Análises Clínicas e Toxicológicas, Faculdade de Farmácia, Universidade Federal do Rio de Janeiro, Rio de Janeiro, Rio de Janeiro, Brazil; 3 Departamento de Bioquímica e Biologia Molecular, Universidade Federal do Paraná, Curitiba, Paraná, Brazil; Stony Brook University, United States of America

## Abstract

*Scedosporium apiospermum* is an emerging fungal pathogen that causes both localized and disseminated infections in immunocompromised patients. Glucosylceramides (CMH, GlcCer) are the main neutral glycosphingolipids expressed in fungal cells. In this study, glucosylceramides (GlcCer) were extracted and purified in several chromatographic steps. Using high-performance thin layer chromatography (HPTLC) and electrospray ionization mass spectrometry (ESI-MS), *N*-2′-hydroxyhexadecanoyl-1-β-D-glucopyranosyl-9-methyl-4,8-sphingadienine was identified as the main GlcCer in *S. apiospermum*. A monoclonal antibody (Mab) against this molecule was used for indirect immunofluorescence experiments, which revealed that this CMH is present on the surface of the mycelial and conidial forms of *S. apiospermum*. Treatment of *S. apiospermum* conidia with the Mab significantly reduced fungal growth. In addition, the Mab also enhanced the phagocytosis and killing of *S. apiospermum* by murine cells. *In vitro* assays were performed to evaluate the CMHs for their cytotoxic activities against the mammalian cell lines L.929 and RAW, and an inhibitory effect on cell proliferation was observed. Synergistic *in* *vitro* interactions were observed between the Mab against GlcCer and both amphotericin B (AmB) and itraconazole. Because *Scedosporium* species develop drug resistance, the number of available antifungal drugs is limited; our data indicate that combining immunotherapy with the available drugs might be a viable treatment option. These results suggest that in *S. apiospermum*, GlcCer are most likely cell wall components that are targeted by antifungal antibodies, which directly inhibit fungal development and enhance macrophage function; furthermore, these results suggest the combined use of monoclonal antibodies against GlcCer and antifungal drugs for antifungal immunotherapy.

## Introduction

The opportunistic pathogen *Scedosporium apiospermum* is present worldwide in plant and soil residues and is involved in a wide range of human infections in both immunocompetent and immunocompromised hosts [1], [2]. In cystic fibrosis patients, *S. apiospermum* is the second most abundant filamentous fungus colonizing the respiratory tract; its colonization frequency ranges from 6.5–10% [3], [4]. Recently, Zouhair *et* *al*. (2013) [5] observed that two other species of the *Pseudallescheria*/*Scedosporium* complex, i.e., *S. aurantiacum* and *P. minutispora* can colonize the respiratory tract of cystic fibrosis (CF) patients. Despite the rising frequency of *Scedosporium*/*P. boydii* infections, the pathogenesis and mechanisms by which these fungi evade host pulmonary defenses and reach other organs are poorly understood [2]. The cell wall glycoconjugates of the *Pseudallescheria*/*Scedosporium* complex have been studied extensively to identify the structures that are critical for fungal physiology and pathogenesis. Elucidation of the primary structures of these glycoconjugates, specifically the monohexosylceramides (CMHs) that function as virulence determinants is important for understanding the mechanisms of fungal pathogenicity. Glucosylceramides (GlcCer) are the main neutral glycosphingolipids expressed in fungal pathogens. GlcCer are bioactive molecules in fungal cells and have several distinct roles. They are associated with fungal growth [6], [7] and morphological transitions in *Cryptococcus neoformans*, *P. boydii*, *Candida albicans*, *Aspergillus fumigatus* and *Collectotrichum gloeosporioides* [8]–[10]. These glycosylated molecules are present in the fungal cell wall and absent from most mammalian cells, and they are excellent targets for the design of new agents that inhibit fungal growth and the differentiation of pathogens. A Mab targeting a fungal glycosphingolipid (Mab to GlcCer) protects mice against lethal *C. neoformans* infection [11]. Because fungal cerebrosides are highly conserved and expressed in almost all known pathogenic species [8], [10], GlcCer-binding antibodies might also be useful for the control of other mycoses.

In this study, we characterized the CMHs in the mycelia of *S. apiospermum*. Structural analyses revealed the presence of the conserved CMHs that were identified in other fungal pathogens [8]. The major CMH is N′-hydroxyhexadecanoyl-1-β-D-glucopyranosyl-9-methyl-4,8-sphingadienine. A Mab against GlcCer was used for immunofluorescence analyses, which indicated that the GlcCer was mainly expressed on the surface of growing cells [12], [13]. The Mab to GlcCer inhibited the growth of *S. apiospermum* conidia and enhanced the antifungal function of murine macrophages. Furthermore, we evaluated the *in* *vitro* susceptibility of *S. apiospermum* to the Mab against GlcCer in combination with the conventional antifungal agents amphotericin B (AmB) and itraconazole.

## Materials and Methods

### Microorganisms and Growth Conditions

The *S. apiospermum* strain was kindly provided by Dr. J. Guarro from Unitat de Microbiologia, Facultat de Medicina e Institut d’Estudis Avançats, Réus, Spain. Cells were maintained on Sabouraud (SAB; 2% glucose, 1% peptone, 0.5% yeast extract) agar slants. Fresh cultures were inoculated in SAB liquid culture medium and incubated for 7 days at 25°C with orbital shaking. Conidia were grown at 30°C on Petri dishes containing SAB agar medium. After 7 days in culture, conidial cells were obtained by washing the plate surface with phosphate-buffered saline (pH 7.2) (PBS; 10 mM NaH_2_PO_4_, 10 mM Na_2_HPO_4_, 150 mM NaCl) and filtering them through gauze to remove hyphal fragments and debris. The conidia were washed three times in PBS (pH 7.2) and counted in a Neubauer chamber.

### Reagents and Cell Lines

MTT [3-(4,5-dimethyl-thiazol-2-yl) 2,5-diphenyl tetrazolium bromide] and paraformaldehyde were obtained from Sigma-Aldrich Co. (St. Louis, MO, USA). Peritoneal macrophages were obtained from male BALB/c mice (4–8 weeks) and maintained in RPMI 1640 medium containing 10% fetal calf serum (FCS). The RAW and A549 cells were maintained in DMEM. In all experiments, the cell counts and viability were determined by trypan blue vital dye exclusion using a hemocytometer. This method yielded conidia that had an initial viability of >95%, as confirmed by plating. Peptidorhamnomannan (PRM) was produced as described [14]. A goat anti-mouse (GAM) IgG was used as an isotype-matched control in all the experiments. For immunofluorescence experiments, an Alexa Fluor 546-conjugated donkey anti-mouse IgG (h4l) was used (Invitrogen Molecular Probes, Carlsbad, CA, USA). Nitric oxide levels were measured using a commercial Griess reagent kit (Promega, Madison, WI, USA).

### Mice

Balb/C mice were obtained from the Universidade Federal do Rio de Janeiro Breeding Unit (Rio de Janeiro, Brazil). The animals were maintained at constant temperature (25°C) with free access to chow and water in a room with a 12-h light/dark cycle. The experiments were approved by the Institutional Animal Welfare Committee of the Federal University of Rio de Janeiro.

### Extraction and Purification of GlcCer from *S. apiospermum*

Intact hyphae of *S. apiospermum* were successively extracted at room temperature using chloroform: methanol at 2∶1 and 1∶2 (v/v) ratios. The extracts were combined and dried, and the crude lipid extract was partitioned as described by Folch *et* *al.* (1957) [15]. The lipids recovered from the Folch lower layer were fractionated on a silica gel column and eluted sequentially with chloroform, acetone and methanol. The acetone and methanol fractions containing glycosphingolipids were then purified further by silica gel column chromatography. This column was sequentially eluted with chloroform/methanol containing increasing concentrations of methanol (95∶5, 9∶1, 8∶2, and 1∶1 v/v) and finally, with methanol. Fractions of 5 ml were collected and analyzed by thin-layer chromatography (TLC), and the plate was developed with CHCl_3_/CH_3_OH/2 M NH_4_OH 40∶10:1 (v/v). The spots were visualized with iodine and by spraying with orcinol/H_2_SO_4_. The chloroform/methanol 8∶2 (v/v) fraction was further purified on Iatrobeads RS 2060 (Macherey & Nagel, Düren, Germany) using the same elution system to obtain a purified glycosphingolipid fraction, as visualized by HPTLC.

### Sugar Analysis

CMH was hydrolyzed with 3 M trifluoroacetic acid at 100°C for 3 h. The resulting monosaccharide was characterized by HPTLC on a silica gel 60 plate developed with n-butanol: acetone: water at 4∶5:1 (v/v) and visualized using the orcinol-sulfuric acid reagent.

### ESI-MS Analysis of *S. apiospermum* Glucosylceramides

The MS analysis was performed in a Quattro-LC electrospray ionization mass spectrometer (ESI-MS) (Waters, Milford, MA, USA) with a triple-quadrupole mass analyzer operating at atmospheric pressure ionization (API) and assisted by a syringe pump (KD Scientific) for sample infusion. Nitrogen was used as the nebulizing and desolvation gas, and the ionization energies were 50 V on the cone and 2 kV on the capillary when operating in the negative ionization mode or 80 V (cone) and 2.5 kV (capillary) when operating in the positive ionization mode. The second stage tandem-MS was obtained by collision-induced dissociation mass spectrometry (CID-MS) using argon as the collision gas and collision energies ranging between 35–60 eV. The samples were prepared in CH_3_OH at 1 mg/ml, then diluted to 0.1 mg/ml in CH_3_OH:H_2_0 (7∶3 v/v) containing 1 mM LiCl for positive ion detection and directly infused into the ESI source at a flow rate of 10 µl/min.

### Cytotoxic Assay of *S. apiospermum* GlcCer

L929 and RAW cells were plated in 96-well polystyrene tissue-culture plates and incubated for 24 h at 37°C and 5% CO_2_ prior to the addition of *S. apiospermum* GlcCer (200, 100, 50, 25, 12.5, 6.2 and 3.1 µg/ml). After a 48-h incubation, the cell viability was measured by the neutral-red dye uptake method [16].

### Release of GlcCer from *S. apiospermum* in the Culture Supernatant

*S. apiospermum* was grown in Sabouraud liquid medium for seven days at room temperature under agitation. The mycelium was filtered out and the supernatant was concentrated and extracted at room temperature with chloroform:methanol at 2∶1 and 1∶2 (v/v) ratios. The crude lipid extract was partitioned as described by Folch *et* *al.* (1957) [15]. The upper layer was carefully removed, and the lipids recovered from the lower layer were concentrated and analyzed by thin-layer chromatography (TLC), and the plate was developed with chloroform: methanol: 2 M ammonium hydroxide 40∶10:1 (v/v). The spots were visualized with iodine and by spraying with orcinol/sulfuric acid.

### Generation of Mabs against *A. fumigatus* GlcCer

Mabs were produced as previously described [9]. Briefly, six week-old female BALB/c mice were immunized intraperitoneally with 50 µg of GlcCer suspended in a 1∶1 (v/v) emulsion of complete Freund’s adjuvant. After 4 weeks, this procedure was repeated using incomplete Freund’s adjuvant. One week later, a final intraperitoneal injection without adjuvant was administered. Three days before fusion, the animals were boosted with an intrasplenic injection of 50 µg of GlcCer. For cellular fusion, the myeloma mouse cell line SP2/0 was mixed in a 1∶5 ratio with spleen cells. Polyethylene glycol (PEG 3000) was slowly added, and after settling for 15 min followed by centrifugation, the cells were resuspended in RPMI-HAT medium supplemented with bovine fetal serum (10%) and plated onto the wells of a flat–bottomed polystyrene microtiter plate. Ten days after the fusion, ELISA was used to select the positive hybridomas. The hybridoma culture whose supernatant contained the highest level of antibodies to GlcCer was expanded and cloned by limiting dilution over a feeder layer of BALB/c-derived macrophages in a 96-well microtiter plate. For preparation of antibodies in higher concentrations, the antibody-producing cells were injected into the peritoneal cavity of BALB/c mice. Antibodies to GlcCer were purified by protein G-affinity chromatography from ascetic fluids and isotyped as IgG2b using the Sigma ISO/2 kit.

### Reactivity of Purified GlcCer with Antibodies to CMH

The reactivity of *S. apiospermum* GlcCer to anti-CMH Mabs was evaluated by ELISA as described by Nimrichter *et* *al*. (2005) [7]. Briefly, *S. apiospermum* CMH was dissolved in ethanol: methanol 1∶1 (v/v), and 1 µg/well was added to a flat-bottomed polystyrene microtiter plate (BD-Falcon, MD, USA). *A. fumigatus* CMH (at 1 µg/well) was used as positive control. The plate was dried and blocked with PBS containing 1% BSA (2 h, 37°C). Decreasing concentrations of the anti-CMH Mab and an unrelated IgG were added, and the plate was incubated at 37°C for 1 h. The plate was washed three times and then incubated with HRP-conjugated anti-mouse IgG (1∶1000 dilution) (Sigma-Aldrich) for 1 h at 37°C. The plate was again washed three times with PBS and the antigen-antibody complexes were detected with 0.04% ortho-phenylenediamine (OPD) in phosphate-citrate buffer at pH 5.0 containing 30 vol. H_2_O_2_. The signal at 490 nm was measured using a spectrophotometer.

### Immunostaining of *S. apiospermum* CMH

CMHs from *S. apiospermum* and *A. fumigatus* were separated by HPTLC, and immunostaining was performed as described by Pinto *et* *al.* (2005) [17]. The CMHs were separated in chloroform: methanol: 2 M NH4OH 40∶10:1(v/v), and the plate was air-dried, soaked in 0.5% poly (isobutyl methacrylate) in chloroform: n-hexane (1∶10, v/v) and then blocked for 2 h with PBS-BSA 1% at 4°C. The plate was then incubated with anti-*A. fumigatus* CMH Mab for 18 h at 4°C followed by sequential incubation with a peroxidase-conjugated anti-mouse IgG (Sigma-Aldrich, 1∶1000 dilution and 0.05% 3,3′-diaminobenzidine (DAB) in PBS and H_2_O_2_ (30 vol.).

### Immunofluorescence Analysis

Mycelial or conidial forms of *S. apiospermum* were fixed in 4% paraformaldehyde cacodylate buffer (0.1 M, pH 7.2) for 1 h at room temperature. The fixed cells were washed twice in PBS and then incubated in PBS containing 1% BSA for 1 h at 37°C. The conidia and mycelium were washed three times with PBS and incubated for 1 h at 37°C with either a Mab to CMH or an isotype-matched control, which was used at a concentration of 50 µg/ml in PBS containing 1% BSA. Cells were washed and incubated in 100 µl of Alexafluor 546 (at 1∶400 dilution) in PBS containing 1% BSA for 1 h at 37°C. After three washes, the cells were suspended in 50 µl of a mounting solution containing 0.01 M *N*-propyl gallate diluted in PBS: glycerol (1∶1, v/v). Ten microliters of the suspension was applied to a microscope slide and examined with an Olympus AX70 fluorescence microscope (Olympus America Inc., Center Valley, PA) using a 620-nm filter and a 100X magnification lens.

### Germination Assay

The germination assay was performed as previously described but with minor modifications [18]. *S. apiospermum* conidia (1×10^5^/ml) were incubated in RPMI 1640 in 24-well plates at 37°C with 50, 25, and 10 µg/ml of the Mab against *A. fumigatus* CMH or PBS. After 12 and 24 h of incubation, the wells were analyzed, and the germinated conidia were counted by optical microscopy. At least 100 conidia per field were counted, and the mean value of three independent counts was calculated. The percent germination was calculated as 100× the ratio of the number of germinated conidia to the total number of cells counted. For viability assays, 3-(4,5-dimethylthiazol-2-yl)-2,5-diphenyltetrazolium bromide (MTT) was added to each well and the plates were incubated at 37°C for 24 h. After incubation, the medium containing MTT was partially removed and DMSO was added to solubilize the MTT formazan product. The absorbance of each well was measured at 570 nm using a spectrophotometer. Both experiments were performed 15 and 24 h.

### Phagocytosis Assays

Phagocytosis assays were performed as described previously [19]. Briefly, peritoneal macrophage cells from BALB/c mice were plated at a concentration of 10^5^ cells per well in 24-well polystyrene cell-culture plates and grown overnight at 37°C in the presence of 5% CO_2_. *S. apiospermum* conidia were collected after 7 days of growth, washed three times with PBS, and 1×10^6^ conidia were incubated with 50, 25, and 10 µg/ml of Mab to CMH for 1 h at 37°C. After washing, the fungal cells were added to the macrophages at a ratio of 5∶1 (conidia: macrophages), and the plates were incubated for 1 h at 37°C in the presence of 5% CO_2_. Samples were prepared in triplicate. The wells were washed with PBS and fixed with Bouin’s fixative solution. The numbers of macrophages and conidia were recorded for each field, and at least 200 macrophages were counted. The phagocytosis index was defined as the ratio of the number of intracellular conidia to the number of macrophages counted.

### Macrophage Effector Functions

The growth of *S. apiospermum* conidia in the presence of Mabs was evaluated by incubating the fungus with either the Mab to CMH or PBS prior to co-culture with macrophages. Washed conidial cells were added to wells containing peritoneal macrophage cells at a ratio of 5∶1 and incubated for 2 h. The cultures were washed with ice-cold PBS, and the macrophages were lysed by adding sterile deionized water. Aliquots were plated on Sabouraud agar plates, and the plates were incubated at 30°C. The percentage of growth was determined as the ratio of the number of CFU for *S. apiospermum* pretreated with Mabs to the number of CFU for untreated conidia.

### *In vitro* Susceptibility of *S. apiospermum* to Antifungals Alone and in Combination with the Anti-GlcCer Mab

*S. apiospermum* conidia were plated at 10^5^ cells per well in 96-well polystyrene tissue-culture plates. Antifungal drugs (itraconazole and amphotericin B) were added in increasing concentrations (0.5 to 16 µg/ml), and 50 µg/ml of Mab to CMH was added. After 24-h incubation at 37°C, the plates were analyzed by measuring the absorbance at 490 nm using a spectrophotometer.

### Statistical Analyses

Statistical analyses were performed using GraphPad Prism version 5.00 for Windows (GraphPad Software, San Diego CA). Unless otherwise noted, one-way analysis of variance using a Kruskal-Wallis nonparametric test was used to compare the differences between groups, and individual comparisons of groups were performed using a Bonferroni posttest. The *t* test was used to compare the number of CFU for different groups. The 90–95% confidence interval was determined in all experiments. Survival results were analyzed by a Kaplan-Meyer test to determine the differences between groups.

## Results

### Structural Analysis of *S. apiospermum* CMHs

**Figure 1:**
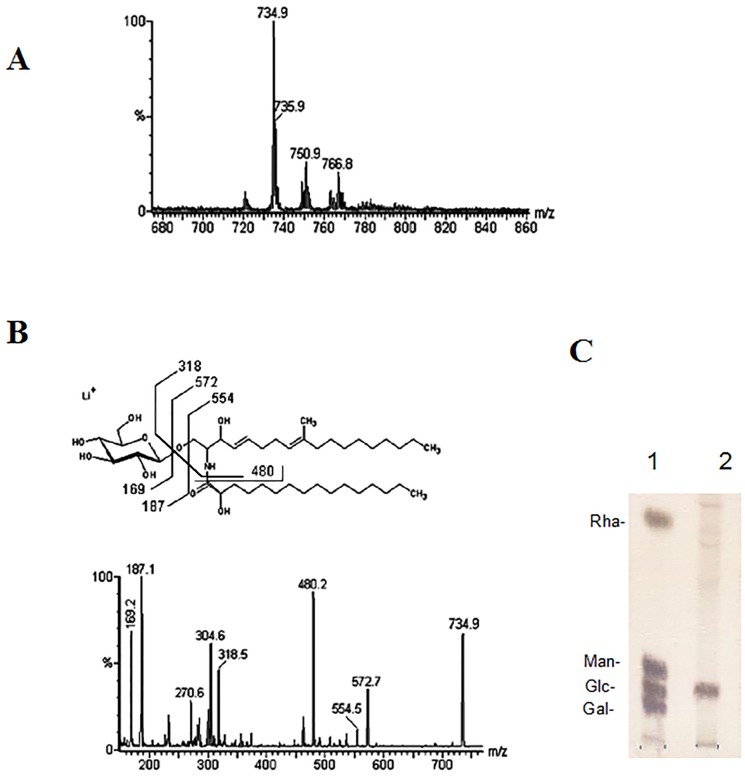
ESI-MS (positive ion mode, Li+ adducts) analysis of the GlcCer species of *S. apiospermum*. (A) MS1 spectrum. (B) ESI-MS2 of the ions species with *m/z* 734.9 observed in (A) and proposed structures for the major GlcCer species in *S. apiospermum.* (C) HPTLC plate of monosaccharides from *S. apiospermum* CMH. 1. Galactose, glucose, mannose and rhamnose standards; 2. Glucose from CMH. The sugars were detected using the orcinol-sulfuric acid spray reagent.

ESI-MS analysis was performed to elucidate the chemical structure of *S. apiospermum* CMHs. A major, lithiated, singly charged ion species at *m/z* 734.9 was observed at the MS1 spectrum **(** **Figure 1A** **)**. When subjected to tandem (MS/MS) fragmentation, this ion species generated fragment ions consistent with the chemical structure of a CMH **(** **Figure 1B** **)**. The loss of 162 units, common to all CMH analyzed and diagnostic of a monosaccharide unit, generated daughter ions at *m/z* 572 [M-hexose+ Li+] and *m/z* 554 [M-hexose-H2O+ Li+] corresponding to the ceramide monolithiated ion from the parental ion at *m/z* 734. The daughter ion at *m/z* 480 is consistent with the loss of a OH-C16 fatty acid. The fragments at *m/z* 187 and 169 confirmed the presence of a hexose. Based on these observations, we concluded that the glycosphingolipid structure consisted of a hexose, a long chain base (9-methyl-4,8-sphingadienine), and a hydroxylated C16∶0 fatty acid **(** **Figure 1B** **)**. To determine the identity of the hexose in the structure, hydrolysis was performed using 3 M trifluoroacetic acid, which revealed that glucose was the sugar constituent of CMH **(** **Figure 1C** **).** Two other minor species were detected at *m/z* 750.9 and 766.8, and they most likely represent differences in hydroxylation and the lengths of the fatty acid chains **(** **Figure 1A** **)**.

### Binding of Mabs to *S. apiospermum* CMH

**Figure 2:**
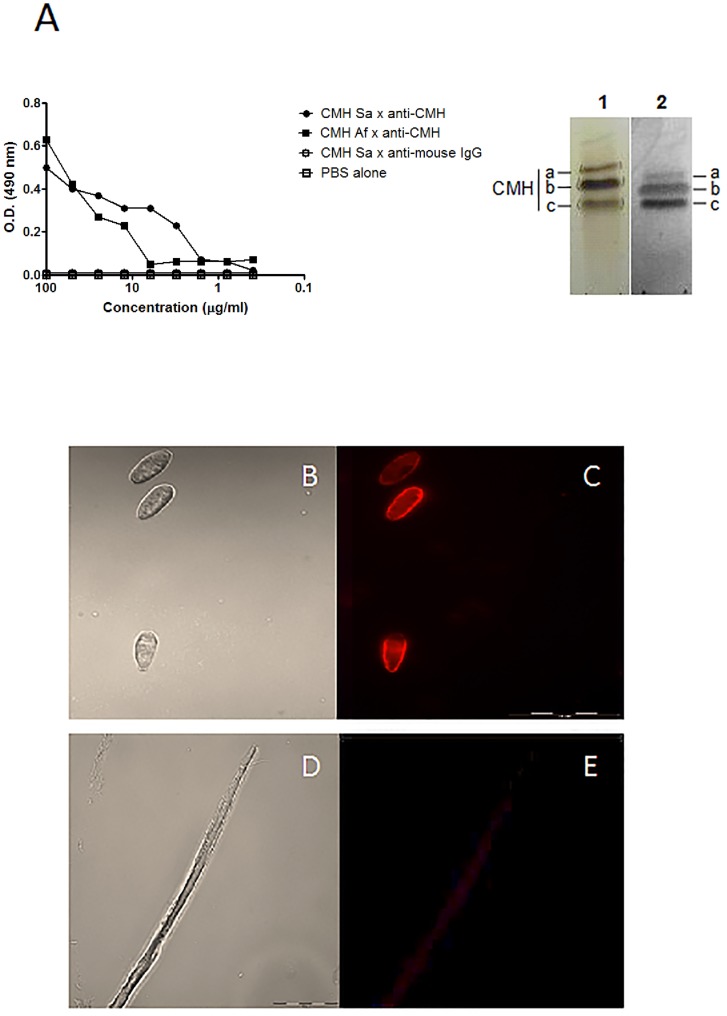
Reactivity of fungal CMH with the anti-CMH MAb. (A) ELISA analysis of the binding of Mabs to *S. apiospermum* CMH. The amount of antibody bound to CMHs was determined by incubation with a rabbit anti-mouse IgG. CMH from *S. apiospermum* (closed circle); CMH from *A. fumigatus* (closed square); CMH from *S. apiospermum* + unrelated antibody (open circle); negative control (open square). Inset: HPTLC of *S. apiospermum* CMH (spots a,b,c), which was developed in CHCl3: MeOH: 2 M NH4OH (40:10:1 v/v). Lane 1: stained with orcinol/ H2SO4; Lane 2: immunostaining with the anti-CMH MAb. Indirect immunofluorescence analysis of conidial (B and C) and mycelial (D and E) forms of *S. apiospermum* using the anti-CMH MAb. B and D: phase contrast; C and E: fluorescence signal.

The specificity of the Mab to *S. apiospermum* CMH was analyzed using ELISA and indirect immunofluorescence and HPTLC immunostaining assays. Indirect ELISA was performed using *A. fumigatus* and *S. apiospermum* cells. The anti-*A. fumigatus* CMH Mab recognized the conidial forms of both fungi **(** **Figure 2A** **)**, indicating the presence of conserved CMH structures on the cellular surfaces of these fungi. The binding of this antibody to the fungal cell surface was also analyzed by fluorescence microscopy. Immunofluorescence analysis of the *S. apiospermum* conidia and mycelium revealed the localization of CMH on the surface of the fungus. As shown in **Figure 2** **B–E,** GlcCer are detectable on the surface of the conidial forms. Analysis of the mycelial forms revealed that the Mab weakly recognized mature hyphae. TLC immunostaining analysis revealed that the anti-*A. fumigatus* CMH Mab recognized bands comigrating with *S. apiospermum* CMHs (spots a, b and c), as revealed by the orcinol reagent **(** **Figure 2A** **, inset)**. These analyses indicate that CMHs are components of the fungal cell wall.

### Cytotoxic Assay of *S. apiospermum* CMH

**Figure 3:**
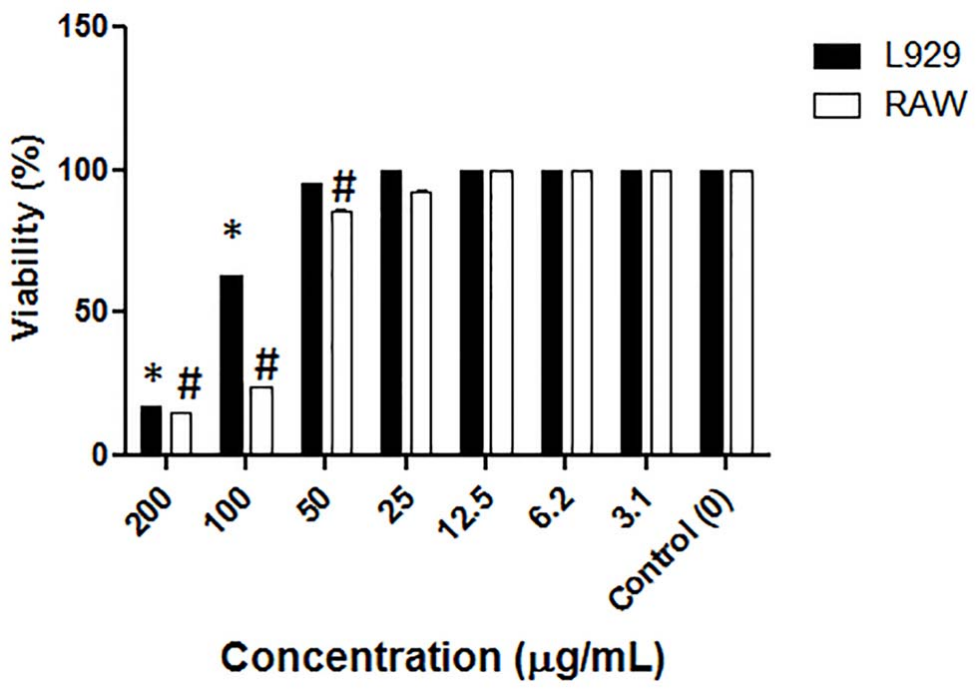
Cytotoxic activity of *S. apiospermum* CMH. Cytotoxic assay of *S. apiospermum* CMH on L929 and RAW cells during 48 h of incubation. Cell viability was measured by adding a neutral-red solution and measuring the absorbance at 490 nm using a spectrophotometer. The presence of *S. apiospermum* CMH in the culture supernatant was analyzed using TLC.

To study the cytotoxicity of the *S. apiospermum* CMH, a cellular cytotoxicity assay was performed using two mammalian cell lines (L.929 and RAW). As shown in **Figure 3**, the CMH from *S. apiospermum* significantly inhibited the growth of both cell lines, and there was a dose-dependent inhibition with increasing concentrations of CMH, with the highest cytotoxic effects observed at 100 and 200 µg/ml of CMH. At 100 and 200 µg/ml of CMH, the viability of L.929 cells was less than 70% and 30%, respectively, compared to control cells. The cytotoxic effect was more pronounced for RAW cells because the cell viability was less than 30% at both 100 and 200 µg/ml of CMH **(** **Figure 3** **)**.

### Release of *S. apiospermum* CMH in the Culture Supernatant

**Figure 4:**
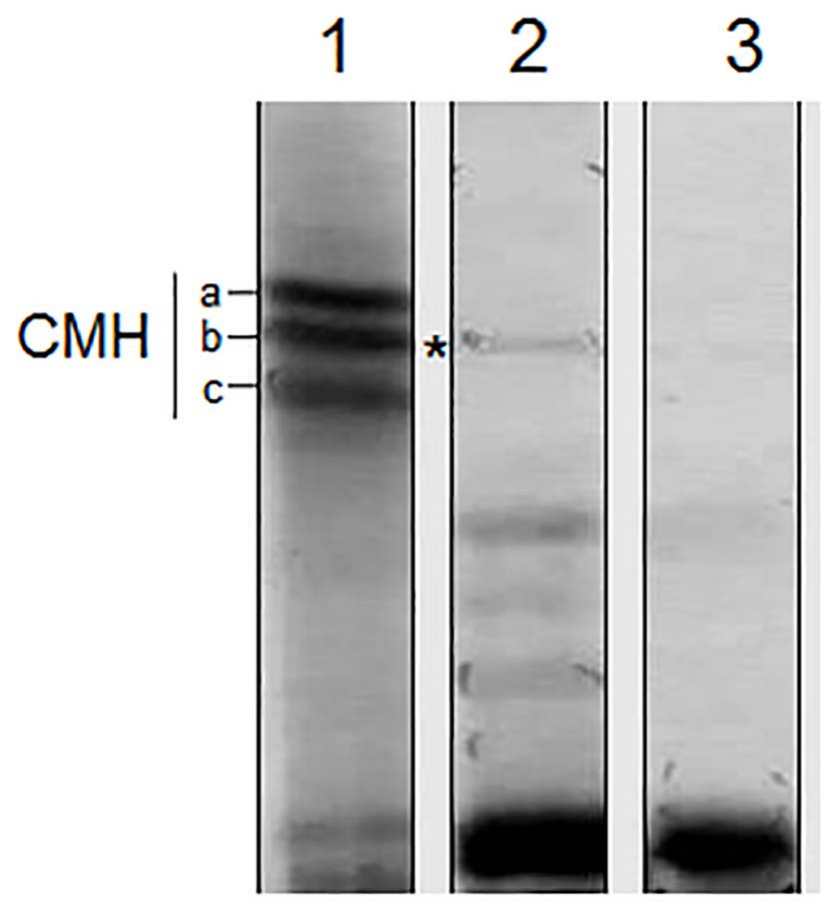
HPTLC analysis of CMH released by *S. apiospermum* in the culture supernatant. Lane 1: Purified CMHs from *S. apiospermum*; Lane 2: CMH released in the supernatant; Lane 3: culture medium supernatant (control). The solvent used was chloroform: methanol: 2 M NH_4_OH (40:10:1 v/v). Detection was performed using iodine and the orcinol-sulfuric acid reagent.

To evaluate the extracellular release of CMH, we tested whether CMH was present in the supernatant of *S. apiospermum* in the culture medium. TLC analysis confirmed the presence of CMH in the extracellular milieu, indicating the possible release of this molecule by the fungus **(** **Figure 4** **)**.

### Influence of Mabs to CMH on *S. apiospermum* Germination and Viability

**Figure 5:**
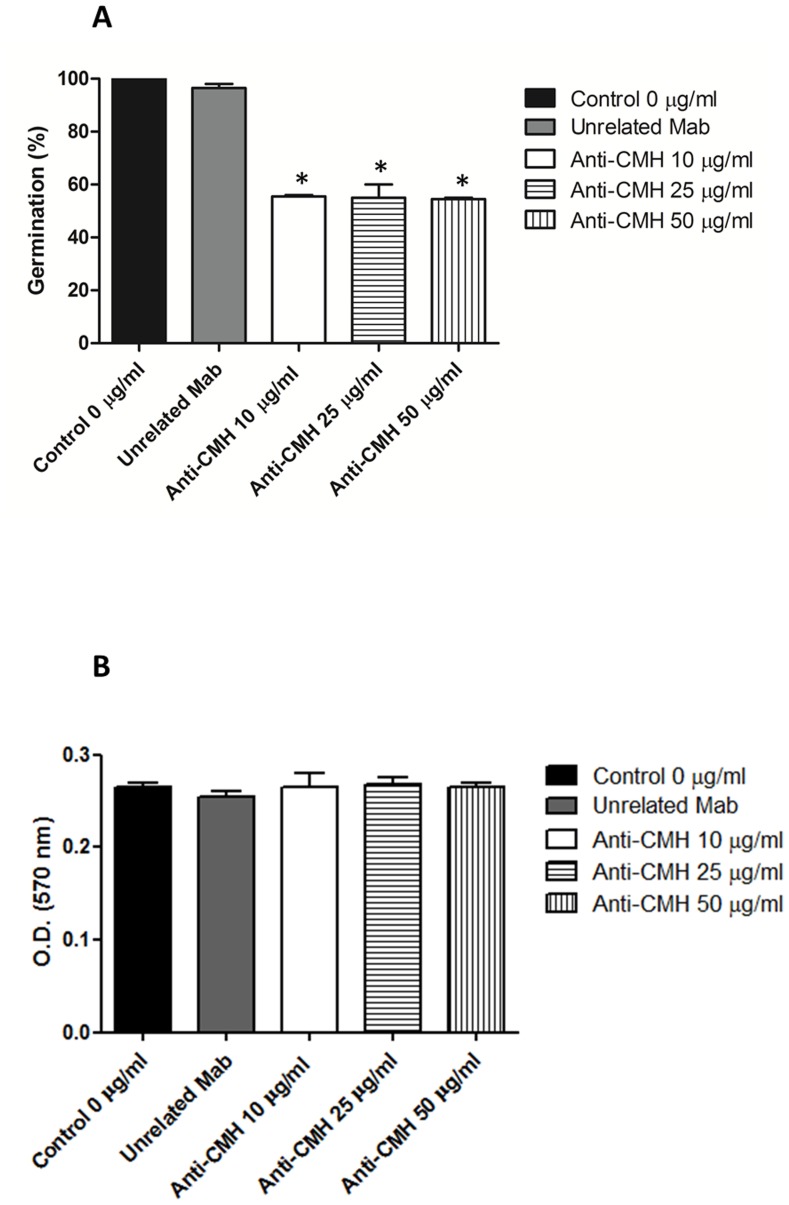
Effect of the anti-CMH Mab on the germination and viability of *S. apiospermum* conidial cells. (A) Germinated conidia in the presence of different concentrations of anti-CMH Mab over 24 h were counted by optical microscopy. At least 100 conidia per field were counted, and the mean value of three independent counts was calculated. (B) Viability assays were performed using *S. apiospermum* conidia and the anti-CMH Mab over 24 h and evaluated using MTT. The absorbance at 570 nm was measured using a spectrophotometer.

MAbs to CMH were used to evaluate the potential of CMHs as targets to inhibit the differentiation of *S. apiospermum.* Conidia were incubated with these antibodies and the process of mycelial differentiation was evaluated at different time points. The germination of *S. apiospermum* conidia was significantly altered by all tested concentrations (50, 25, and 10 µg/ml) of the Mabs to CMH. The conidia-mycelium transition decreased by approximately 50% after 15-h incubation **(data not shown)** and by 40% after 24-h incubation **(** **Figure 5A** **)**, compared to the controls, which were not treated with the antibodies (p<0.05). These results indicate that binding of anti-CMH Mabs to conidial cells impedes the conidia-mycelium transition. However, viability analyses revealed that the decreased germination is not followed by an alteration in cell viability. After 15 **(data not shown)** and 24 h of incubation **(** **Figure 5B** **)**, no significant change in cell viability was observed compared to the controls (p>0.05). These results suggest that the reduction of germination is caused by a fungistatic process rather than by cell death.

### Influence of the Anti-CMH Mab on the Interaction between *S. apiospermum* Conidia and Peritoneal Macrophages

**Figure 6:**
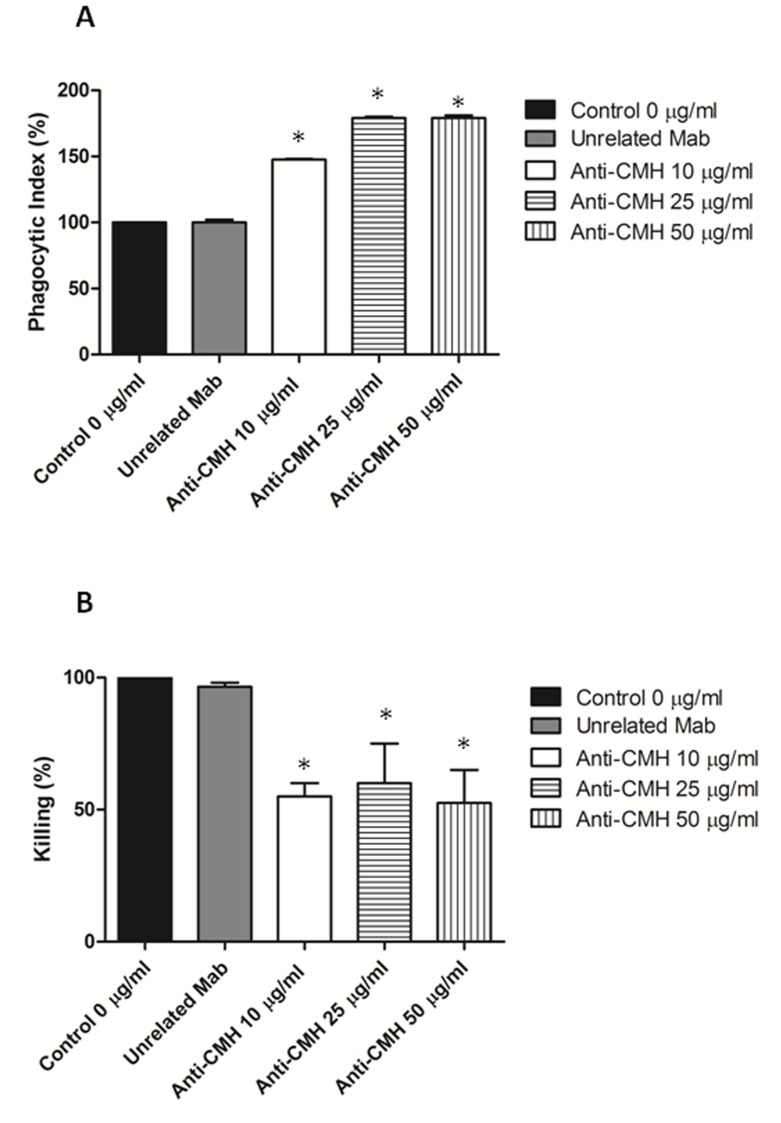
MAb to CMH affect phagocytosis. Influence of the Mab to CMH on the phagocytosis of *S. apiospermum* conidia cells by peritoneal macrophages (A) and on phagocyte antimicrobial activity. Fungal cells treated with the MAb against GlcCer were more efficiently internalized, as demonstrated by the phagocytic indices. (B) Microbial killing by macrophages was enhanced after treatment with antibodies to CMH.

To evaluate whether the anti-CMH Mab influences the interaction between fungal cells and the host immune cells, fungal cells were treated with the anti-CMH Mab before interaction with macrophages. Treatment of fungal cells with the antibody to CMH resulted in a significant increase of phagocytosis by peritoneal macrophages compared to the controls (p<0.05), as shown in **Figure 6A** **.** In addition, fungal cells coated with the Mab against CMH were more efficiently killed by macrophages than untreated conidia **(** **Figure 6B** **)**; these data indicate that apart from its direct antifungal action, the anti-CMH Mab also enhances the antimicrobial activity of host macrophages.

### *In vitro* Susceptibilities of *S. apiospermum* to Itraconazole and AmB Alone and in Combination with Anti-CMH Mabs

**Figure 7:**
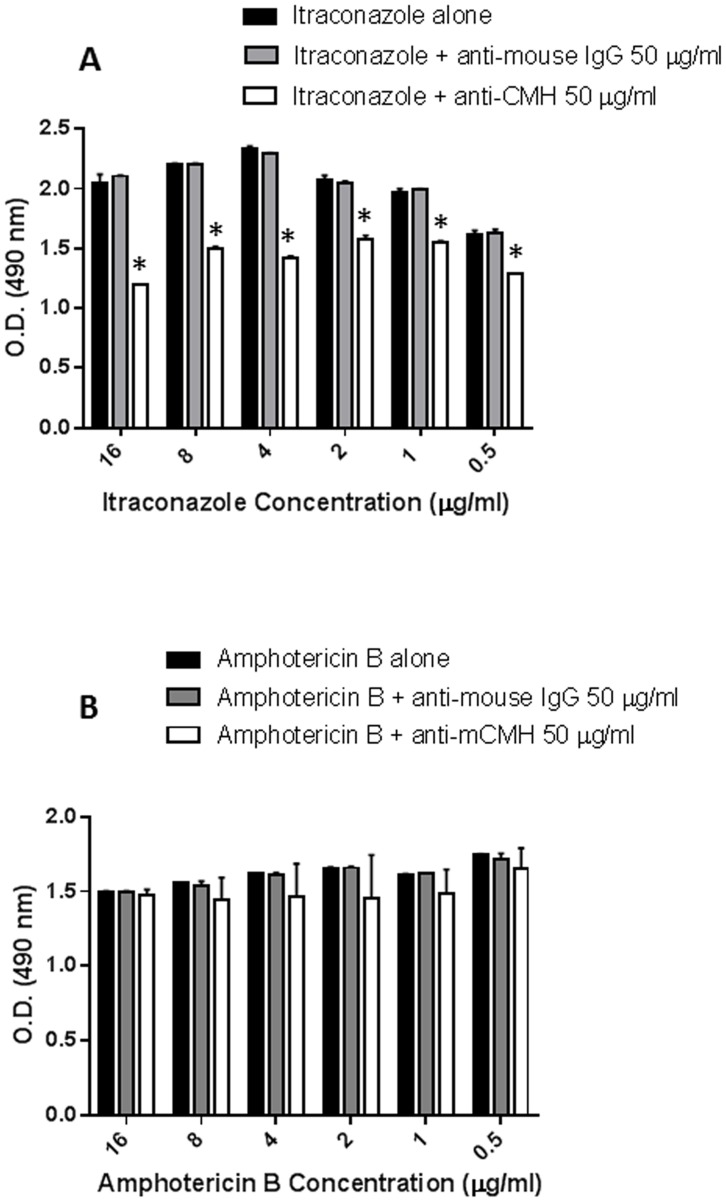
Synergistic effect of combining the anti-CMH Mab with antifungal drugs. The viability of *S. apiospermum* conidia was analyzed in the presence of the anti-CMH Mab (50 µg/ml) and different concentrations (0.5 -16 µg/ml) of itraconazole (A) or amphotericin B (B). The values shown are means of three independent experiments.

The *in* *vitro* antifungal effect of 0.5–16 µg/ml of itraconazole and AmB on *S. apiospermum* conidia was tested either alone or in combination with the anti-CMH Mab. The anti-CMH MAb increased the viability inhibition of all concentrations of itraconazole **(** **Figure 7A** **)**. No synergistic effect of the antibody was observed on Amphotericin B activity **(** **Figure 7B** **).**

## Discussion

*Scedosporium* species are involved in a wide range of human infections, especially in immunocompromised patients [20]. In these diseases, cerebral abscesses are relatively frequent [21], reflecting the neurotropic character of these fungi. A typical manifestation of these fungal diseases is the near-drowning syndrome [21], when patients develop cerebral abscesses weeks to months after the near-drowning incident [22]. In cystic fibrosis patients, *Scedosporium* species are among the most common fungal colonizers of the respiratory tract but rarely become invasive [3]. The majority of *Scedosporium* isolates display multiple antifungal resistance patterns. Because *Scedosporium* species do not have a normal MIC/MEC distribution, prediction of the antifungal susceptibility of a single strain is difficult, but the species have differing trends of susceptibilities to antifungal compounds [23]. The increasing frequency and high mortality rates of invasive infections caused by *Scedosporium* species necessitate the search for new treatment strategies [24]. Combination therapy might be an alternative to monotherapy for patients with invasive infections that are difficult to treat, such as those caused by the majority of *Scedosporium* isolates, which have multiple antifungal resistance patterns; combination therapy might also be useful for those patients who fail to respond to standard treatment [25]–[27]. It is important to identify new alternatives for the prevention or treatment of fungal infections. The study of additional targets of antifungal agents is extremely relevant, especially for targets associated with the fungal cell wall, which is a vital structure for fungal growth and differentiation. Therefore, cerebrosides might be promising targets for antifungal agents because they are vital for fungal viability [6], [9], [12], [28] and are highly conserved among fungal pathogens [8].

In this study, glycosphingolipids were isolated from the mycelia of *S. apiospermum* and purified to homogeneity using Iatrobeads chromatography. The structures of the glycosphingolipids were determined by chemical and spectrometric methods. The major species of glycosphingolipids produced by *S. apiospermum* was identified as N-2′-hydroxyhexadecanoyl-1-β-D-glucopyranosyl-9-methyl-4,8-sphingadienine (Figure 1). We previously described this structure for molecules in *Fusarium* sp [29], *P. boydii* [28] and the conidial and mycelial forms of *Fonsecaea pedrosoi* [7], [12]. We also detected a minor species appearing at *m/z* 750 [M+ Li+]. Similar results were obtained in the analysis of glycosphingolipids from *Cladosporium resinae* [30], a saprophytic species commonly found as a contaminant in fuel oil storage tanks [30], [31]. Sclerotic cells of *F. pedrosoi* also express the ion at *m/z* 750, which is most likely generated from a ceramide with an LCB containing one or more hydroxyl groups [7], [12].

The immunofluorescence experiments using a monoclonal anti-CMH antibody suggested that the GlcCer have different distributions in the mycelial and conidial forms. The immunofluorescence staining pattern of the conidial forms with the anti-CMH Mab used in this study is consistent with the model that GlcCer are distributed evenly throughout the fungal surface and are accessible to the anti-CMH Mab, whereas in the mycelial forms, the GlcCer localization does not permit interaction with this antibody.

Weak staining of the mycelial forms of *P. brasiliensis* and hyphae of *A. fumigatus* with an IgG2a monoclonal anti-glucosylceramide antibody (MEST-2) was previously observed [32]. The lower reactivity of the mycelial surface with these antibodies could be because mycelial cells might express lower amounts of GlcCer, or these cells might have a different type of cell wall assembly in which the conserved GlcCer are less accessible to antibodies.

The surface distribution of GlcCer in *S. apiospermum* is suggestive of an involvement of these molecules in fungal growth. We showed that incubation of conidia with the monoclonal antibody to CMH significantly reduced cell growth. However, the decreased germination was not followed by an alteration in cell viability. These results suggest that the reduction of germination is caused a fungistatic effect rather than by cell death. Our previous studies using *C. albicans*, *C. neoformans*, *C. gloeosporioides*, *P. boydii* and *F. pedrosoi* are consistent with other results [6], [9], [12], [28]. The mechanism by which anti-CMH antibodies inhibit fungal growth and/or differentiation are yet unclear; however, it is possible that CMHs are associated with enzymes involved in the hydrolysis and synthesis of the fungal cell wall during differentiation and division [33].

In this study, conidial cells treated with the anti-CMH antibody were more effectively phagocytosed than the control PBS- or anti-mouse IgG-treated conidia. The phagocytic indices of conidial cells treated with the anti-CMH antibody were approximately 50% or higher than those of the PBS- or anti-mouse IgG- treated fungi; the phagocytic index increased in a dose-dependent manner with the three antibody concentrations used. In addition, macrophages killed fungal cells coated with the anti-CMH Mab more efficiently than untreated conidia; this effect was observed at the three antibody concentrations used. Therefore, apart from its direct antifungal action, the anti-CMH Mab also enhances the antimicrobial activity of host macrophages.

Previously, we obtained similar results using *F. pedrosoi,* the most frequent etiological agent of chromoblastomycosis. Nimrichter *et* *al*. (2004) [12] demonstrated for the first time that anti-CMH antibodies have direct antifungal action and help host cells to eliminate the internalized *F. pedrosoi* conidial cells. However, pretreatment of conidia with the anti-CMH Mab increased the phagocytic index of *F. pedrosoi* by murine macrophages and increased the antifungal activity of these phagocytes by six-fold. This observation indicates that CMHs are accessible to opsonizing antibodies at the cell wall; this might be an additional mechanism by which these antibodies promote host defense during infection by *F. pedrosoi* and other fungal pathogens such as *S. apiospermum*, which express CMHs. A similar mechanism was proposed to explain the protective ability of antibodies against glucuronoxylomannan, a capsule polysaccharide in *C. neoformans* [34].

In this study, we provide the first evidence for the *in* *vitro* cytotoxicity of a GlcCer from *S. apiospermum*. The GlcCer (at 100 to 200 µg/ml) had a dose-dependent inhibitory effect on the viability of both tested mammalian cell lines L.929 (mouse fibroblasts) and RAW (macrophage-like cells). Recently, four glucocerebrosides were isolated from the sea cucumber *Cucumaria frondosa*, and the *in* *vitro* cytotoxic effect of the molecules on Caco-2 colon cancer cells was evaluated [35]. An inhibitory effect on cell viability (27–32%) was observed when the cells were treated with 400 µg/ml of cerebrosides. In this study, we also observed the spontaneous release of GlcCer in the culture. However, because the amount of GlcCer was very low, it could not be quantified to estimate its physiological concentration. The release of CMH *in* *vivo* from *S. apiospermum* might influence the interaction of this fungus with the invaded host organism. Previous studies have shown that GlcCer is required for the survival of *C. neoformans* in the extracellular environment of the host [36].

*Scedosporium* species have low *in* *vitro* and *in* *vivo* susceptibilities to traditional antifungal drugs [23], [37], [38]. Several studies have shown that antifungal drugs such as amphotericin B, nystatin, fluconazole, ketoconazole, itraconazole and terbinafine have very weak effects on *S. apiospermum in* *vitro* [22]. Some studies have shown a low level of antifungal activity by itraconazole [21], [23], [37], [38]. However, because of the emergence of itraconazole-resistant *Scedosporium* strains, voriconazole has emerged as a potent antifungal drug used to treat disseminated Scedosporiosis [39]. The new triazoles such as voriconazole, ravuconazole and posaconazole have some *in* *vitro* activities against *P. boydii*; however, not all strains of this fungus responded equally to voriconazole. The limited choice of antifungal drugs and the increased resistance of *Scedosporium* species to the commonly used antifungal agents indicate the necessity for other therapeutic strategies. However, there are limited data on the clinical efficacy of combination therapy for *Scedosporium* infections [22], [40], [41].

Because fungi of the *Pseudallescheria/Scedosporium* complex have low susceptibilities to antifungal drugs, several studies have analyzed the combination of different drugs. The combination of amphotericin B with either fluconazole or micafungin had a strong synergistic effect [22], [26], [27], [42]; however, the mechanism of this synergy is unclear. The antifungal activity of micafungin is through inhibition of (1,3)-β-d-glucan synthase and the subsequent disruption of fungal cell wall synthesis. This activity may enhance the action of other, less-active antifungals such as amphotericin B or itraconazole and supports the combined use of micafungin with azoles in future *in* *vitro* and *in* *vivo* studies [22].

Cuenca-Estrella *et* *al*. (2008) [43] showed that Amphotericin B alone inhibited *Scedosporium* strains poorly, but synergistic effects have been observed *in* *vitro* with the combined use of Amphotericin B and various azoles and other agents.

Some studies have also observed a synergy between certain antifungal agents and antibodies. For instance, the combination of caspofungin and anti-β-glucan intensifies the damage caused to hyphae by polymorphonuclear cells [44].

Therefore, combination therapy using an antifungal drug with anti-GlcCer antibodies instead of other chemical drugs might be an alternative treatment method.

The anti-GlcCer Mab used in this study protects mice against lethal *C. neoformans* infection [13]. This protective activity is associated with enhanced phagocytosis and killing of the fungi [6], [12]. The antifungal activity of antibodies to GlcCer and the conserved distribution of these glycolipids in fungal pathogens suggest that the GlcCers might be promising targets for the design of vaccines that elicit the production of protective antibodies.

The MAb that targets β-1,7mannotriose, which is a component of the *C. albicans* phosphomannan, is protective against disseminated forms of the disease, and the antifungal activity is associated with enhanced phagocytosis and killing of the fungus [45]. The combined use of this MAb with AmB enhances its therapeutic efficacy against disseminated Candidiasis. The combination has a synergistic effect, and the use of a reduced AmB concentration might be beneficial because this would decrease the severe side effects of the antibiotic [46].

In this study, we evaluated the *in* *vitro* susceptibility of *S. apiospermum* to AmB and itraconazole alone and in combination with the anti-GlcCer Mab. The best results were obtained by combining itraconazole with the anti-GlcCer Mab. However, no synergistic effect was observed between the anti-CMH Mab and AmB. This synergistic effect might be specific because an anti-mouse IgG (unrelated antibody) did not have a protective effect. Itraconazole is more effective than Amphotericin B in cases of *Scedosporium* infections. But recently we are having some problems because of the emergence of Itraconazole-resistant Scedosporium strains. It can be a possible explanation for a synergy between CMH antibody and Itraconazole, but not Amphotericin B. Many species in the *Pseudallescheria/Scedosporium* complex show intrinsic resistance to Amphotericin B. Therefore, these studies indicate that combining itraconazole with the anti-CMH Mab might represent a solution to the problem of limited antifungal drug choices because of the drug resistance of *Scedosporium* species. To further analyze the potential use of this drug and anti-CMH Mab combination, *in* *vivo* studies of scedosporiosis are necessary. Such studies will elucidate which *in* *vitro* results are relevant to *in* *vivo* effects.
